# Resolving the Family Tree of Placental Mammals

**DOI:** 10.1371/journal.pbio.0040111

**Published:** 2006-03-14

**Authors:** Liza Gross

Natural selection was particularly inventive in the appendage and accessory department during the evolution of placentals—an expansive category of mammals that bear live, fully developed offspring. A placental might sport webbed wings, a prehensile tail, flippers, fangs, tusks, cloven hooves, paws, claws, floppy ears, horns, or any number of other specialized structures. While such morphological characteristics shed light on evolutionary relationships, they can also confound classifications because animals might independently acquire the same traits without sharing a common ancestor.

With an ever-growing repository of genome sequence data, scientists have increasingly turned to molecular techniques to help resolve evolutionary relationships. Several recent molecular analyses offer support for placing recent placentals into four major groups: Afrotheria (mostly African species, including elephants and aardvarks), Xenarthra (New World species such as armadillos and sloths), Laurasiatheria (includes carnivores, whales, and horses), and the newly reclustered Supraprimates (includes rodents and primates). Supraprimates and Laurasiatheria are further grouped together as sister taxa in a larger group called Boreotheria. Molecular approaches have also tried to resolve the hotly debated issue of where to draw the base of the placental tree, though no consensus has emerged. These studies arrived at these conclusions by analyzing different datasets of nuclear and mitochondrial genes under different models of DNA sequence evolution.

But such molecular approaches have their own limitations, as genomes can also contain confounding features (called homoplasies), similar elements that look alike but do not represent common ancestry. In a new study, Jan Ole Kriegs, Jürgen Schmitz, and their colleagues used a different molecular strategy to infer the evolutionary history of placentals, relying on retroposons to signal kinship. Unlike mitochondrial or nuclear genes, retroposons are virtually free from homoplasies. They are reliable markers for inferring evolutionary history, the researchers explain, because their integration into the genome is random—making it highly unlikely for the same element to integrate independently into a conserved region of the genome (called an orthologous position) in two different species. In addition to finding “significant support” for the previously identified divisions, they offer strong support for placing Xenarthra—armadillos and their kin—at the base of the placental tree.

Using specialized computer software, Kriegs et al. searched the mouse, dog, and human genome databases for the presence (or absence) of retroposons. From the 237 candidates identified in the scan, they designed PCR primers (a technique to identify and generate sufficient amounts of specific sequence for analysis) to amplify the equivalent sequences from organisms representing each placental superorder. When size differences between amplified and original sequences (indicating the presence or absence of a retroposed element) occurred within orthologous genome sites, the researchers repeated the analysis with loci from different taxa. (For example, an element might be present in all boreotherian species, but absent in afrotherians and xenarthrans, which diverged before the insertion occurred.) Twenty-eight loci showing size shifts within orthologous sequences were identified for further sequence analysis.

Kriegs et al. next studied the presence/absence patterns of these loci to determine how the various placental representatives were related. This analysis yielded markers that provided solid evidence for the divergence of several superordinal groups, as well as the base branch on the placental tree. Four markers occupied the same orthologous location in every species sampled except for the opossum, demonstrating the power of retroposons to reveal evolutionary splits, even as long as 100 million years ago. Eleven markers were present in all sampled supraprimates and laurasiastherians but not in Afrotheria or Xenarthra, supporting the supergroup Boreotheria. The separate laurasiatherian and supraprimate classifications were also reinforced by the identification of markers found exclusively within both groups.

Evidence that Xenarthra represents the first split in the placental tree comes from the finding that two markers are present in both Boreotheria and Afrotheria but not in Xenarthra. This suggests that Xenarthra represents a sister group to all the other placental mammals (collectively referred to as Epitheria)—a hypothesis proposed by classical morphological taxonomists.

Interestingly, other molecular techniques have come to different conclusions, with a 2001 molecular study of nuclear and mitochondrial DNA reporting that Afrotheria was likely the earliest diverging group. But Kriegs et al. make a strong case that retroposons provide a reliable metric for identifying the likely inhabitants of the basal branch of the placental tree—Xenarthra. With this technique added to their genomics toolbox, scientists can continue to investigate this and other questions concerning placental evolution as more xenarthran and afrotherian sequence data become available. By combining high-throughput bioinformatics with high-throughput diagnostic lab techniques, this study provides a valuable framework for homing in on the true genetic footprints of evolutionary history.

## 

**Figure:**
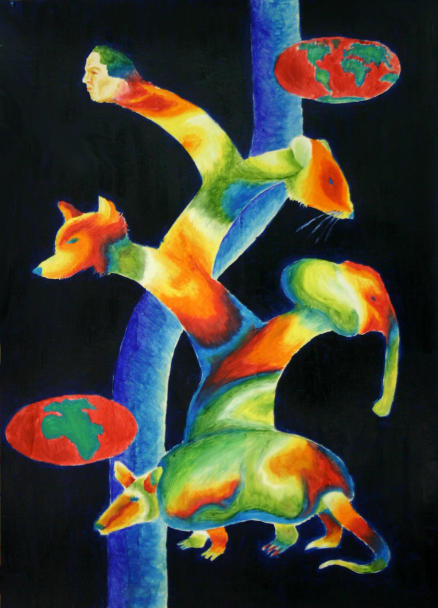
In this painting of the evolution of placental mammals, the blue time line connects two globes of different earth ages symbolizing the time scale of evolution. Major divergences of mammalian lineages are symbolized by members of crown groups. (Painting: Tomas Schmitz)

